# Biogenic Synthesis of Silver Nanoparticles Using *Pantoea stewartii* and *Priestia aryabhattai* and Their Antimicrobial, Larvicidal, Histopathological, and Biotoxicity Potential

**DOI:** 10.3390/bioengineering10020248

**Published:** 2023-02-13

**Authors:** Jeyaraj John Wilson, Thangamariyappan Harimuralikrishnaa, Thangavel Sivakumar, Shunmugiah Mahendran, Ponnirul Ponmanickam, Ramasamy Thangaraj, Subramanian Sevarkodiyone, Naiyf S. Alharbi, Shine Kadaikunnan, Baskar Venkidasamy, Muthu Thiruvengadam, Rajakumar Govindasamy

**Affiliations:** 1 Department of Microbiology, Ayya Nadar Janaki Ammal College, Sivakasi 626124, India; 2 Department of Zoology, Ayya Nadar Janaki Ammal College, Sivakasi 626124, India; 3 Department of Botany and Microbiology, College of Science, King Saud University, Riyadh 11451, Saudi Arabia; 4 Department of Oral and Maxillofacial Surgery, Saveetha Dental College and Hospitals, Saveetha University, Chennai 600077, India; 5 Department of Applied Bioscience, College of Life and Environmental Sciences, Konkuk University, Seoul 05029, Republic of Korea; 6 Department of Orthodontics, Saveetha Dental College and Hospitals, Saveetha Institute of Medical and Technical Sciences, Saveetha University, Chennai 600077, India

**Keywords:** silver nanoparticles, *Pantoea stewartii* H2, *Priestia aryabhattai* H3, biocontrol agent, larvicidal activity, antimicrobial, histopathology, toxicity

## Abstract

In recent years, green nanotechnology has gained considerable importance for the synthesis of nanoparticles due to its economic viability and biosafety. In the current study, silver nanoparticles were synthesized using two bacterial isolates, H2 and H3, which were isolated from soil samples collected from the Western Ghats, Tamil Nadu, and identified at the species level as *Pantoea* *stewartii* (H2) and *Priestia* *aryabhattai* (H3) by sequencing their 16s rRNA genes. Intracellularly synthesized silver nanoparticles were characterized by UV–visible spectroscopy, Fourier transform infrared spectroscopy, atomic force microscopy, and particle size analysis. AFM studies show that both of the bacterial synthesized Ag NPs were circular-shaped and disaggregated, with an average size distribution of 4 nm for *Pantoea* *stewartii* and 3.6 nm for *Priestia* *aryabhattai*. Furthermore, their larvicidal activity, antimicrobial, histopathological, and biotoxicity effects were determined. The synthesized Ag NPs exhibited potent larvicidal activity against fourth instars of *Ae.* *aegypti*, *An.* *stephensi*, and *Cx.* *quinquefasciatus* exposed to a 50 µg/mL concentration for 24 h based on their LC_50_ and LC_90_ values. Histopathological studies of the affected mosquito larvae clearly show damage to the epithelial cells, food bolus, basement membrane, muscles, and midgut parts. The maximum antimicrobial activity of *Priestia* *aryabhattai*-synthesized Ag NPs was observed for *Streptomyces varsoviensis* MTCC-1537, and that of *Pantoea stewartii*-synthesized Ag NPs was against *Escherichia coli* MTCC-43. The toxicity test on non-target organisms such as *Artemia nauplii* and zebrafish embryos indicates no visible abnormalities or mortality after their exposure for 48h. It is concluded that silver nanoparticles can easily be synthesized using *Pantoea stewartii* (H2) and *Priestia aryabhattai* (H3) as capping and reducing agents. Silver nanoparticles showed potent larvicidal activities and could potentially be used in integrated vector control programs because they are safe for other inhabitants of the same aquatic environment as mosquito larvae.

## 1. Introduction

Mosquitoes, a subfamily of the insect family Culicidae, are the most frequent vectors of deadly diseases, such as malaria, dengue fever, chikungunya, filariasis, and yellow fever, which are transferred through the biological transmission of blood by mosquitoes feeding on susceptible vertebrate hosts. About 390 million people worldwide are infected with dengue, of which 96 million may develop clinical symptoms, making dengue fever an epidemic in tropical and subtropical areas in recent years [1]. The prevention of dengue fever is now a top priority for health officials and medical personnel everywhere [2,3,4]. Despite developing a vaccine, there is no particular treatment for dengue [5]. More than 120 million people worldwide have been affected by the principal lymphatic filariasis mosquito vector, *C. quinquefasciatus* [6,7]. Synthetic pesticides, especially pyrethroids and organophosphates, are frequently used to control increased mosquito infestations [8].

However, there is still no effective eco-friendly alternative to eradicate the mosquito population. In the present scenario, nanoparticles have been suggested as an emerging technology and provide various benefits over traditional approaches. Nanoparticles interact more frequently with other molecules because of their high surface areas and their extremely small sizes [9]. Strongly charged nanoparticles can deliver drugs to the desired location by remaining inside blood vessels for longer [10].

The poisonous and unfriendly nature of various techniques, such as solvothermal, sol–gel, chemical reduction, laser ablation, and inert gas condensation, should be avoided [11,12] due to the expense and safety risks associated with nanoparticles produced through chemical and physical processes. Therefore, the green nanoparticle synthesis method is the focus of our investigation. The chemical synthesis of metallic nanoparticles is less environmentally friendly than green synthesis [13,14]. The Ag-NP biological strategy makes it safer to exploit natural resources, such as plants, bacteria, algae, fungi, and actinomycetes [15].

There is evidence in the literature that biological materials, such as microbes, plant extracts, milk, and panchakavya, can synthesize nanoparticles [16,17,18,19,20,21,22]. Numerous studies have reported the potential application of these biologically synthesized Ag NPs [23,24,25,26]. Bacteria, cellulose, enzymes, and plant leaves are used as templates for synthesizing nanoparticles [27].

Using biological agents such as microbes to synthesize nanoparticles (NPs) has drawn much attention as an alternative to traditional techniques. In addition to being simple, inexpensive, and environmentally benign, microorganism-mediated NP synthesis does not use any hazardous chemicals. Bacteria are widely used in NP synthesis because they are replicable and viable nanofactories for various NPs, including gold, zinc, silver, and selenium nanoparticles [28,29]. Some bacteria have specialized biological mechanisms for creating nanoparticles (NPs) by detoxifying metal ions, primarily through metal ion biosorption and bioaccumulation [30]. Studies have shown that the capacity of bacteria to withstand metallic ions plays a critical role in their ability to produce NPs. Specifically, bacteria use the structure of their cell membrane and proteins released by the cell to reduce metallic ions into harmless nanoparticles (NPs), protecting them from the ions’ detrimental effects [31]. Numerous functional groups in the bacterial cell membrane serve as ligands for metallic ions, so it is often the location where NPs are formed [32].

The ability of nanoparticles to act as antimicrobial agents for different pathogens is made possible by the tiny size difference between bacteria and nanoparticles. Since nanomaterials can easily encapsulate biological molecules to be incorporated with such systems, it makes it easier to develop effective and novel biomedical applications for use as promising materials in nanomedicine. Compounds intended to kill bacteria locally or slow down their growth with the least toxic effects on the surrounding tissue are collectively referred to as having antimicrobial activity. Nanomaterials serve as drug delivery vehicles, antimicrobial agents, vaccine delivery vehicles, and immunomodulators [33].

Antibiotic resistance among bacteria has recently increased, posing a more significant threat to people. Nanoparticles are new agents with a vast surface area for their small size. These physical and chemical characteristics make them exceptionally capable of battling pathogenic bacterial species and penetrating cell walls; this has been confirmed in many experiments. It has been noted that Ag NPs inhibit both Gram-positive and Gram-negative microorganisms [34]. Ag NPs may emit Ag+, which is harmful to bacteria, and Ag NPs have bactericidal characteristics [35]. In topical treatments, food packaging, and wound dressings, Ag NPs have been employed as antibacterial agents [36,37]. The unrestricted use of conventional antibiotics also plays a significant role in developing drug-resistant genes and developing resistance, which contributes to multidrug resistance [38]. As a result, the usage of Ag NPs can potentially address the problem of antibiotic resistance [39].

The present study focused on synthesizing Ag NPs using bacterial isolates and assessing their larvicidal effects against the mosquito vectors *Culex quinquefasciatus*, *Anopheles stephensi,* and *Aedes aegypti*. Furthermore, histological and stereomicroscopic research was used to investigate the target site of Ag NPs on mosquito larvae. Ag NPs were also assessed for their antibacterial potential against harmful microbes. Furthermore, *Artemia salina* and embryonic zebrafish were used to determine the impact of Ag NPs on non-target organisms.

## 2. Results

### 2.1. Molecular Identification of Bacteria

**Figure 1:**
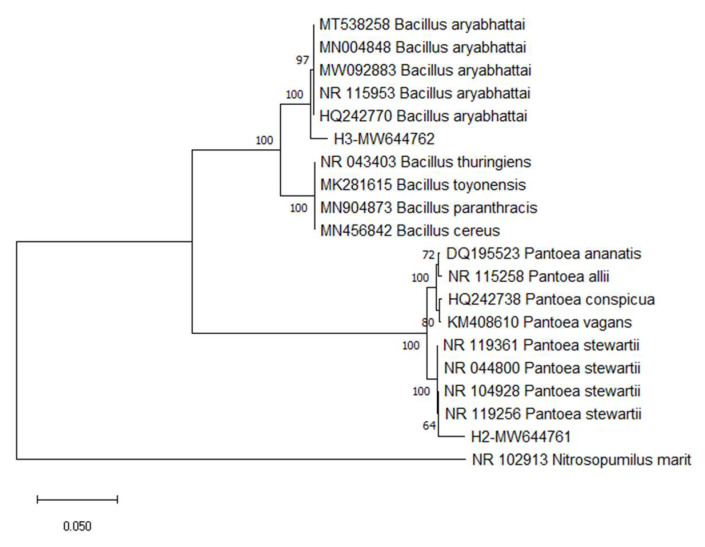
The neighbor-joining tree represents the phylogenetic placement of strains H2 and H3 with their reference strains.

The findings led to strains H2 and H3 being categorized as *Bacillus* and *Actinomyces* species. Isolated bacteria were recognized at the molecular level by 16S rRNA gene sequencing for further verification. The 16S rRNA gene was sequenced after the genomic DNA was extracted, amplified, and purified. Based on a similarity search and phylogenetic analysis, the sequence was deposited in Genbank, and the accession numbers (MW644761 for strain H2 and MW644762 for strain H3) were determined. *Pantoea stewartii* and *Priestia aryabhattai* were the closest to 100% similarity to H2 and H3, respectively (Figure 1). The *Pantoea stewartii* strain and the *Priestia aryabhattai* strain were determined to be strains H2 and H3 based on conventional and molecular identification data.

### 2.2. Biosynthesis of Silver Nanoparticles

**Figure 2:**
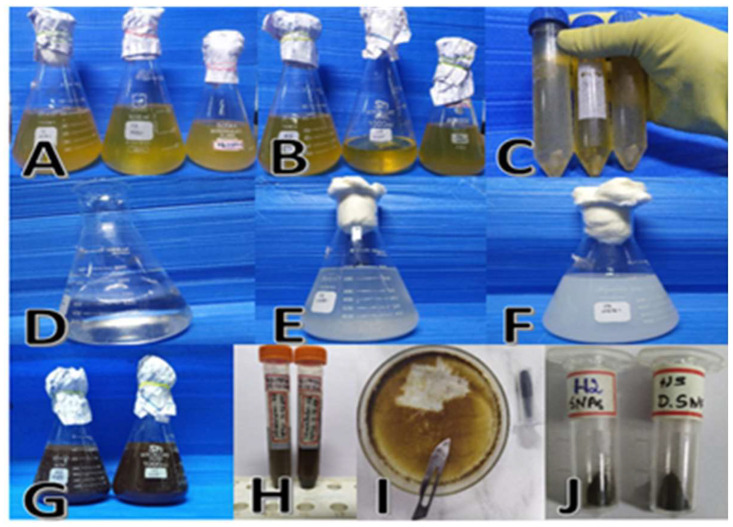
Synthesis of silver nanoparticles using *Pantoea stewartii* and *Priestia aryabhattai* ((**A**)—bacterial broth culture of *Pantoea stewartii*; (**B**)—*Priestia aryabhattai*; (**C**)—bacterial pellet; (**D**)—distilled water; (**E**)—bacterial pellet and distilled water; (**F**)—bacterial pellet, distilled water, and 1 mM silver nitrate; (**G**)—broth changed to dark brown color, indicating silver nanoparticle synthesis; (**H**)—separated nanoparticle; (**I**)—dried silver nanoparticles; (**J**)—powder form of silver nanoparticles).

Bacteria were isolated from a soil sample and characterized. For using bacterial isolates for silver nanoparticle synthesis, biomasses were prepared. The microbial cultures were grown aerobically with continuous agitation at 200 rpm using an orbital shaker at 37 °C. After 24 h of growth, the microbial biomass was collected and centrifuged at 10,000 rpm for 10 min at 4 °C. Silver nitrate (1 mM concentration) was added to culture pellet aliquots containing solutions of *Pantoea stewartii* (H2) and *Priestia aryabhattai* and incubated at 60 °C under dark conditions. The color changed from pale white to brown, indicating the formation of Ag NPs. The intracellular synthesis of silver nanoparticles was induced in the pellet of the culture using silver nitrate, and it was observed to change in color from pale white to dark brown (Figure 2).

### 2.3. Characterization of Silver Nanoparticles

#### UV–Visible Spectroscopy Analysis

**Figure 3:**
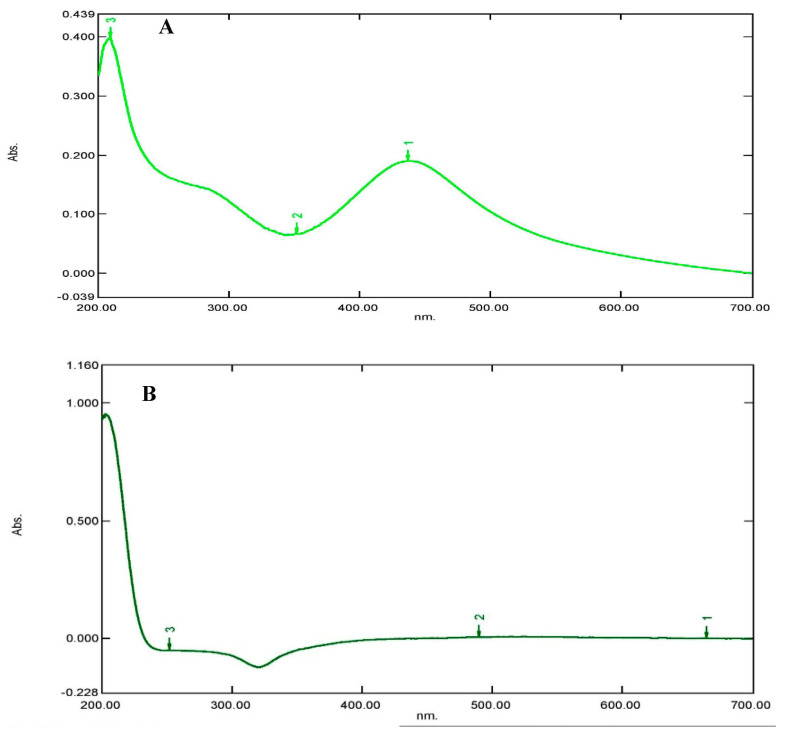
UV–visible spectra of (**A**) *Pantoea stewartii*- and (**B**) *Priestia aryabhattai*-synthesized silver nanoparticles.

The absorption spectra of the *Pantoea stewartii*- and *Priestia aryabhattai*-synthesized silver samples were characterized by UV–visible spectroscopy. An environmentally benign method of creating nanoparticles is biosynthetic nanotechnology. For the intracellular production of stable Ag NPs, *Pantoea stewartii* and *Priestia aryabhattai* may be crucial biological elements. The reduction of silver ions to silver nanoparticles was confirmed by the visible color change of the reaction mixture from white to brown. The brown color could be due to the excitation of surface plasmon vibrations, typical of silver nanoparticles. The silver colloidal substance’s dark brown color indicates that the silver nanoparticles have surface plasmon resonance. The UV–visible absorption spectrometric analysis of *Pantoea stewartii* (437 and 351 nm) and *Priestia aryabhattai* (490 nm and 664 nm) showed smooth and narrow absorption bands (Figure 3).

### 2.4. FT-IR Analysis of Pantoea stewartii- and Priestia aryabhattai-Synthesized Silver Nanoparticles and Bacterial Pellets

**Figure 4:**
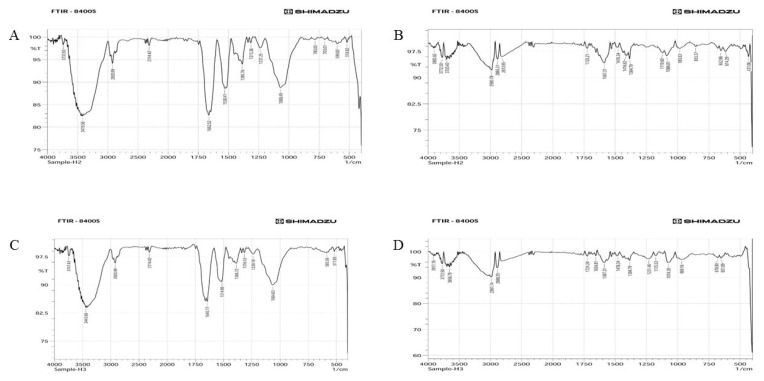
FT-IR spectra of (**A**) *Pantoea stewartii*-synthesized silver nanoparticles, (**B**) pellet of *Pantoea stewartii*, (**C**) *Priestia aryabhattai*-synthesized silver nanoparticles, and (**D**) pellet of *Priestia aryabhattai*.

To determine the potential mechanism underlying the synthesis of silver nanoparticles and to provide details on the functional groups, FT-IR spectroscopy measurements were conducted (Figure 4). Different types of functional groups are formed with the addition of the synthesized silver nanoparticles. The FT-IR results of *Pantoea stewartii*- and *Priestia aryabhattai*-synthesized silver nanoparticles show various functional groups from 518.82 cm^−1^ to 3733.93 cm^−1^ and from 517.85 cm^−1^ to 3747.43 cm^−1,^ respectively. The FT-IR results of the culture pellets of *Pantoea stewartii* and *Priestia aryabhattai* show different functional groups from 431.06 cm^−1^ to 3720.43 cm^−1^ and from 651.89 cm^−1^ to 3772.5 cm^−1,^ respectively. Comparing the FT-IR spectra of the bacterial pellets and synthesized Ag NPs, groups such as alkenes, alcohols, aromatic amines, nitro compounds, and halo compounds were only present in bacterially synthesized Ag NPs. The presence of these active functional groups indicates the formation of silver nanoparticles.

### 2.5. Atomic Force Microscopy

**Figure 5:**
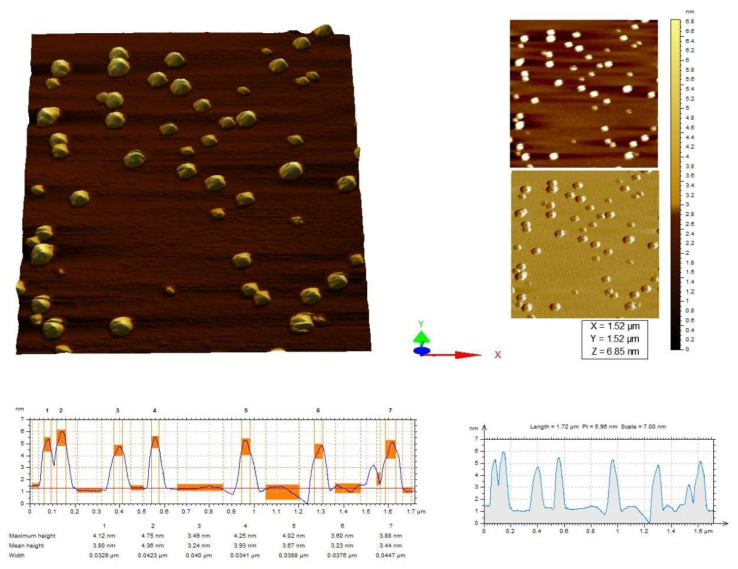
AFM images (2D and 3D) of *Pantoea stewartii*-synthesized Ag NPs.

**Figure 6:**
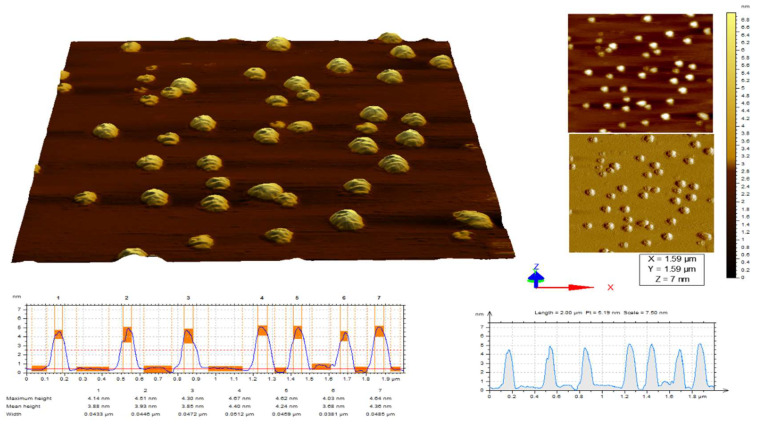
AFM images (2D and 3D) of *Priestia aryabhattaii*-synthesized Ag NPs.

AFM was performed for the morphological study of *Pantoea stewartii*- and *Priestia aryabhattai*-synthesized silver nanoparticles. Both bacterially synthesized Ag NPs were circular-shaped and disaggregated with a size distribution in the range of 4 nm (*Pantoea stewartii*) and 3.6 nm (*Priestia aryabhattai*) (Figure 5 and Figure 6).

### 2.6. Larvicidal Bioassay

#### 2.6.1. Larvicidal Activity

The larvicidal efficacy of *Pantoea stewartii*- and *Priestia aryabhattai*-synthesized silver nanoparticles (Ag NPs) was tested against the fourth-instar larvae of *Ae. aegypti, Cx. quinquefasciatus*, and *Anopheles stephensi* at different concentrations (10, 20, 30, 40, 50 μg/mL). It was observed that *Pantoea stewartia*- and *Priestia aryabhattai*-synthesized Ag NPs showed a considerable dose-dependent mortality rate.

#### 2.6.2. Larvicidal Activity of *Pantoea stewartii*-Synthesized Silver Nanoparticles

The effect of Ag NPs on the mortality rates of fourth-instar larvae of *Aedes aegypti, Culex quinquefasciatus,* and *Anopheles* was monitored for 24 h. The results show that Ag NPs exhibited dose-dependent activity, and 100% mortality was achieved in a 50 µg/mL concentration of Ag NPs in all three mosquito species. The bacterially synthesized Ag NPs showed strong larvicidal activity based on their LC_50_ and LC_90_ values. *Pantoea stewartii*-synthesized Ag NPs were the most toxic to *Cx. quinquefasciatus*, with an LC_50_ value of 14.829 (8.539–25.754), an LC_90_ value of 43.575 (25.091–75.677) µg/mL, and a χ^2^ value of 0.864, followed by *An. stephensi*, with values of 20.977 (12.399–35.488), 62.550 (36.972–105.824) µg/mL, and 0.34, and *Ae. aegypti*, with values of 30.584 (17.072–54.789), 106.354 (59.367–190.530) µg/mL, and 0.762, respectively. The percentage of mortality, LC_50_, LC_90_ (LUL-UCL), intercept, χ^2^ value, and regression equation of *Pantoea stewartii*-synthesized Ag NPs against the three mosquito species are tabulated in Table 1.

#### 2.6.3. Larvicidal Activity of *Priestia aryabhattai*-Synthesized Silver Nanoparticles

The effect of Ag NPs against fourth-instar larvae of *Aedes aegypti, Culex quinquefasciatus,* and *Anopheles stephensi* shows a nearly 100% mortality rate over 24 h. *Priestia aryabhattai*-synthesized Ag NPs exhibited dose-dependent activity, and the highest mortality rate was observed at a 50 µg/mL concentration. Strong larvicidal activity was demonstrated by the synthesized Ag NPs, with LC_50_ and LC_90_ values of 14.015 (6.652–29.529) and 66.544 (31.584–140.202) µg/mL for *An. stephensi*, 20.668 (10.985–38.886) and 79.911 (42.473–150.347) µg/mL for *Ae. aegypti*, and 10.736 (5.186–22.228) and 44.394 (21.443–91.91) µg/mL for *Cx. quinquefasciatus*, respectively (Table 2). *Priestia aryabhattai*-mediated Ag NPs had the most toxic effect on *Culex quinquefasciatus*, followed by *Anopheles stephensi* and *Aedes aegypti*.

### 2.7. Histopathological and Stereomicroscopic Analysis

**Figure 7:**
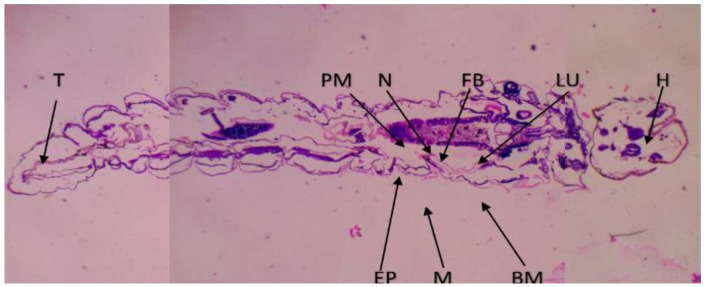
Histopathological images of fourth-instar larvae of non-treated *Ae. aegypti* larvae (epithelial cells—EP; peritrophic membrane—PM; food bolus—FB; basement membrane—BM; muscles—M; nucleus—N; LU—gut lumen).

**Figure 8:**
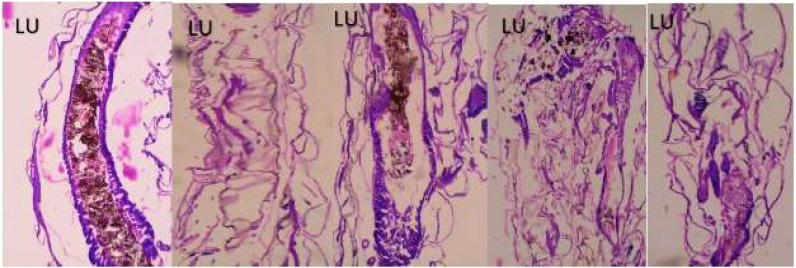
Longitudinal section and midgut lumen of an *Aedes aegypti* larva exposed to different concentrations of *Pantoea stewartii*-mediated silver nanoparticles.

**Figure 9:**
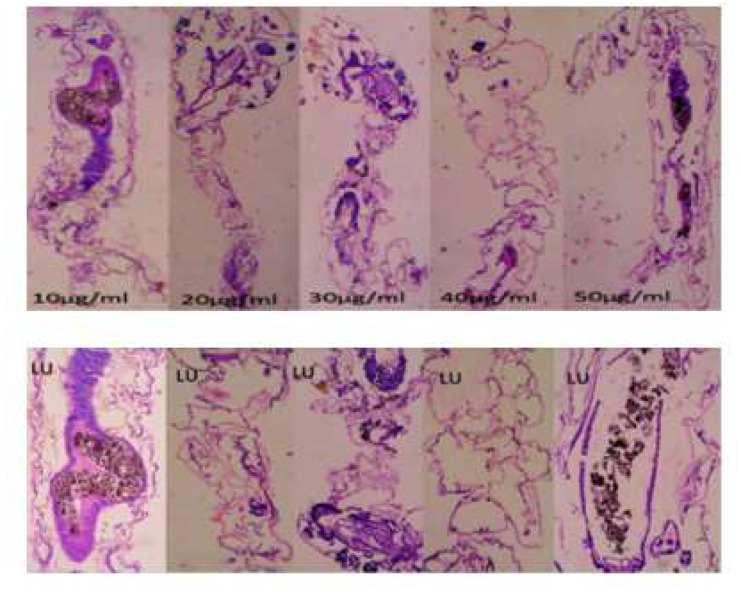
Longitudinal section and midgut lumen of an *Aedes aegypti* larva exposed to a concentration of *Priestia aryabhattai*-mediated silver nanoparticles.

**Figure 10:**
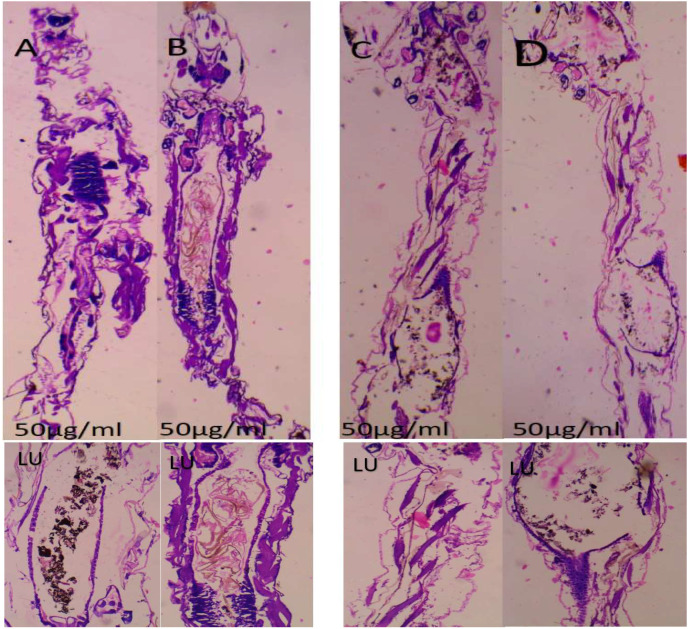
(**A**,**B**) Longitudinal section and midgut lumen of *Anopheles stephensi* larva exposed to 50 μg/mL concentration of *Pantoea stewartii*- (**A**) and *Priestia aryabhattai*-mediated (**B**) silver nanoparticles; (**C**,**D**) longitudinal section and midgut lumen of *Culex quinquefasciatus* larva exposed to 50 μg/mL concentration of *Pantoea stewartii*- (**C**) and *Priestia aryabhattai*-mediated (**D**) silver nanoparticles.

After the bacterially synthesized silver nanoparticle treatment, larval body parts, such as the epithelial cells, food bolus, basement membrane, muscles, and midgut, were damaged. The damage to the larval bodies depended on the concentration of nanoparticles. The larval gut lumen was mainly injured, and it was focused on separately. The current study showed that the midgut epithelial cells of treated larvae had altered histological structures, while control larvae showed no such changes (Figure 7). After being exposed to bacterially synthesized Ag NPs, the fourth-instar larvae of *Aedes aegypti*, *Culex quinquefasciatus*, and *Anopheles stephensi* displayed varying degrees of apical enlargement into the gut lumen, a reduction in intercellular connections, and nuclei and brush border degeneration (Figure 8, Figure 9 and Figure 10).

### 2.8. Antimicrobial Activities of Silver Nanoparticles Synthesized by Microorganisms

Wells of 6 mm diameter were cut in sterile nutrient agar plates using a gel puncture and swabbed with 100 μL of the test organisms (108 CFU/mL). Each well was loaded with different concentrations (30, 50, and 80 μg/mL) of synthesized Ag NPs, with chloramphenicol as a control, and incubated at 37 °C ± 0.2 °C. After 24 h incubation, the zones of inhibition (cm) were measured.

#### 2.8.1. Antimicrobial Activity of *Priestia aryabhattai* (H3)-Synthesized Ag NPs

The antimicrobial activity of *Priestia aryabhattai*-synthesized Ag NPs was tested against MTCC-1537 *Streptomyces varsoviensis*, MTCC-655 *Streptococcus pneumoniae*, MTCC-160 *Penicillium chrysogenum*, and MTCC-3160 *Staphylococcus aureus*. A confluent lawn of microbial cultures was created by swabbing 24-hour-old test microbial cultures onto a nutrient medium. The plates were incubated at 27 °C for 24 h. The zones of inhibition were measured and are tabulated in Table 3.

#### 2.8.2. Antimicrobial Activity of *Pantoea stewartii* (H2)-Synthesized Ag NPs

The antimicrobial activity of *Pantoea stewartii*-synthesized Ag NPs was tested against MTCC-1537 *Streptomyces varsoviensis*, MTCC-160 *Penicillium chrysogenum,* MTCC-3160 *Staphylococcus aureus,* MTCC-1688 *Pseudomonas aeruginosa,* MTCC-733 *Salmonella enterica serovar typhi*, MTCC-1973 *Aspergillus flavus,* MTCC-451 *Vibrio parahaemolyticus*, and MTCC-43 *Escherichia coli.* The 24-hour-old test microbial culture was swabbed onto a nutrient medium to form a confluent lawn of microbial cultures, and the plates were incubated at 27 °C for 24 h. The zones of inhibition were measured and are tabulated in Table 4. *Pantoea stewartii*-synthesized Ag NPs showed the strongest antimicrobial activity against *E. coli*, followed by MTCC-1537 *Streptomyces varsoviensis* and MTCC-160 *Penicillium chrysogenum*.

### 2.9. Non-Target Bioassay on A. salina

*Artemia* swallows all particles smaller than 50 microns because of its typical nonselective filter-feeding behavior. In addition to concentration, the quantity of aggregation is also influenced by the different concentrations of nanoparticles each animal consumes. In this study, various Ag NP concentrations were used to treat *Artemia nauplii*, namely, 10 µg/mL, 20 µg/mL, 40 µg/mL, 60 µg/mL, and 90 µg/mL, for 48 h. The study revealed that the animals in the control group did not exhibit any signs of aggregation. Ag NPs synthesized by *Pantoea stewartii* and *Priestia aryabhattai* showed less toxicity to the non-target species *A. nauplii* than to mosquitoes (less than 50%).

#### 2.9.1. Toxicity Assay of *Pantoea stewartii*-Mediated Ag NPs

No mortality was observed in the control or groups treated with *Pantoea stewartii*-mediated Ag NPs at lower concentrations (10 and 20 µg/mL). The LC_50_, LC_90_, and χ^2^ values were 63.44 (38.484–104.579), 157.095 (95.297–258.968), and 0.619 (Table 5).

#### 2.9.2. Toxicity Assay of *Priestia aryabhattai*-Mediated Ag NPs

Mortality was undetectable in the control and groups treated at lower concentrations (10 and 20 µg/mL) of *Priestia aryabhattai*-mediated Ag NPs. Higher amounts (90 µg/mL) resulted in 67% mortality. The values of LC_50_ and LC_90_ were 74.595 (42.455–131.065) and 206.893 (117.752–363.517), respectively. No behavioral changes were visible after treatment with *Pantoea stewartii* - and *Priestia aryabhattai*-mediated Ag NPs (Table 6).

### 2.10. Toxicity Studies on Embryonic Zebrafish

**Figure 11:**
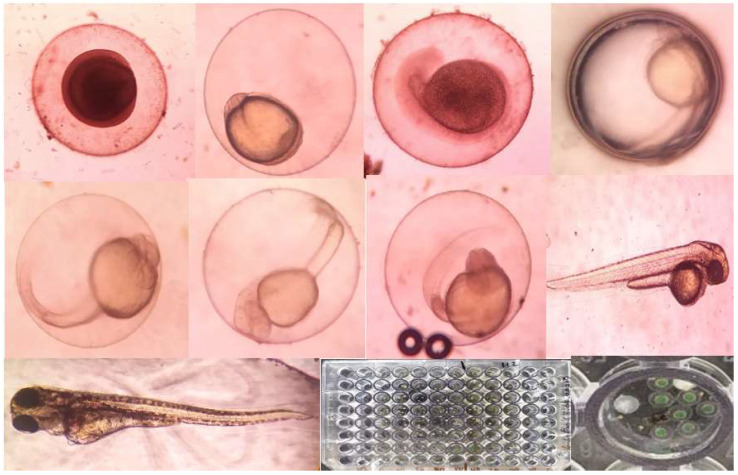
Control images of eggs and embryos of Zebrafish (not exposed to silver nanoparticles) at 120 hpf.

The percentages of mortality and malformation for embryos exposed to *Pantoea stewartii*- and *Priestia aryabhattai*-synthesized Ag NPs are displayed in Table 7 and Table 8, respectively. The black color indicates mortality. Zebrafish embryos were exposed to Ag NP concentrations of 10, 20, 30, and 50 µg/mL. The lowest embryonic toxicity in the presence of the chorion was found with Ag NPs synthesized by both bacterial species at the highest concentration (50 µg/mL). No incidence of mortality was observed at a lower concentration. After exposure to Ag NPs at 50 µg/mL, over 70% of the embryos had non-malformed yolk sac edema. Ag NPs synthesized by *Priestia aryabhattai* and *Pantoea stewartii* were less harmful to non-target zebrafish eggs than to mosquitoes (Figure 11).

### 2.11. Toxicity Studies on Embryonic Zebrafish Exposed to Pantoea stewartii-Mediated Ag NPs

**Figure 12:**
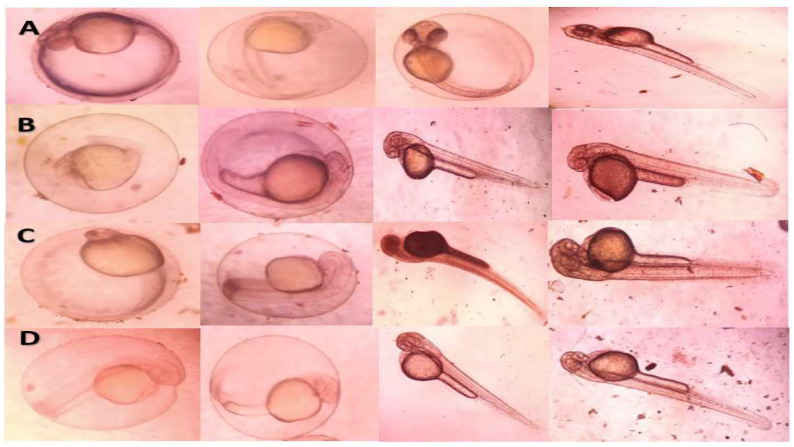
Representative images of embryos exposed to *Pantoea stewartii*-synthesized silver nanoparticles (Ag NPs) at 120 hpf: (**A**)—10 µg/mL; (**B**)—20 µg/mL; (**C**)—30 µg/mL; and (**D**)—50 µg/mL.

At 48 h after treatment, *Pantoea stewartii*-mediated Ag NPs showed low (less than 70%) embryo mortality. There was no mortality in controls and groups treated with lower concentrations (10 and 20 µg/mL). The LC_50_, LC_90_, and χ^2^ values were 299.187 (76.007–1,177.70), 2,038.019 (517.748–8022.291), and 0.92 (Table 7 and Figure 12).

### 2.12. Toxicity Studies on Embryonic Zebrafish Exposed to Priestia aryabhattai-Mediated Ag NPs

**Figure 13:**
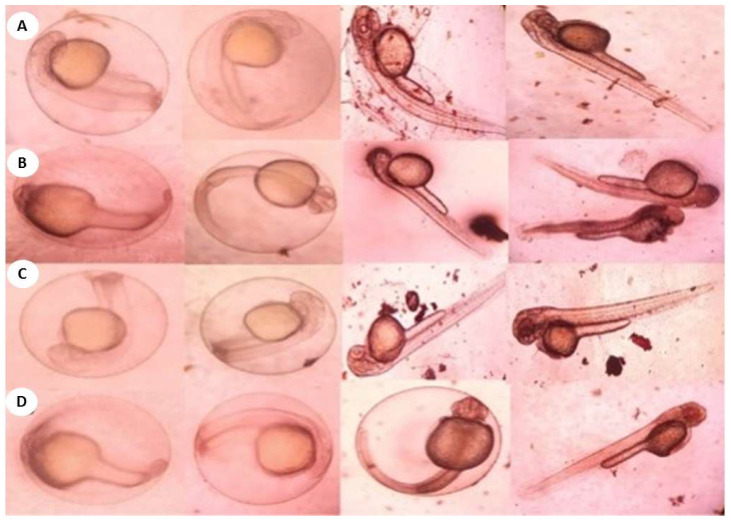
Representative images of embryos exposed to *Priestia aryabhattai*-synthesized silver nanoparticles (Ag NPs) at 120 hpf. (**A**)—10 µg/mL; (**B**)—20 µg/mL; (**C**)—30 µg/mL; (**D**)—50 µg/mL.

*Priestia aryabhattai*-mediated Ag NPs resulted in minimal (less than 20%) mortality in embryos 48 h after treatment. There was no mortality in controls and groups treated with lower concentrations (10 and 20 µg/mL). The LC_50_ and LC_90_ values were 101.032 (47.534–214.74) and 331.537 (155.983–704.668), and the χ^2^ value was 0.716 (Table 8 and Figure 13).

## 3. Discussion

The present study concentrated on the environmentally friendly method of microbially manufacturing silver nanoparticles, which involves nanotechnology. Silver nanoparticles (Ag NPs) were synthesized using bacterial strains of *Pantoea stewartii* (H2) and *Priestia aryabhattai* (H3) and evaluated for their larvicidal activity against *Aedes aegypti*, *Anopheles stephensi*, and *Culex quinquefasciatus* mosquito. The soil contained a variety of bacterial communities, and a spread plate technique was used to serially dilute the soil sample and plate it on nutritional agar. At 37 °C, the plates were incubated. The growth was monitored for 24 h. To obtain pure isolates, only distinct colonies grown on the nutrient agar were selected and subcultured on separate nutrient agar mediums. Similarly, Kalaimurugan et al. [40] obtained *Pseudomonas fluorescens* YPS3 soil bacteria from the soil (Yercaud hills), which are known to vary in diversity using the spread plate technique. A bacterium was recovered from the rhizospheric soil by Park et al. [41], and using 16S rRNA sequencing, the bacterium was determined to be *Bacillus aryabhattai* strain SRB02.

The pure isolates obtained were characterized to determine their morphological, physiological, and biochemical nature. Purified bacterial cultures were used for the green synthesis of silver nanoparticles using the intracellular method, with silver nitrate as a substrate. Bacterial biomass (2 g) was incubated with 100 mL of a 1 mM AgNO_3_ solution overnight at 60 °C, pH 10. After incubation, the color changed from pale white to brown. The color change from colorless to light brown indicated the formation of Ag NPs in the reaction mixture. High pH is an important factor in bacteria-mediated Ag NP production. High pH also activates reductases among oxidoreductase enzymes. Das et al. [42] studied bacterial phenotypic, biochemical, and, finally, molecular characteristics and found that the culture was a *Bacillus* strain. They used the bacterial culture supernatant for the extracellular synthesis of silver nanoparticles by mixing it with a filter-sterilized AgNO_3_ solution at a final concentration of 1 mM while focusing on the biological synthesis of silver nanoparticles.

The synthesized silver nanoparticle characterization was performed using UV-vis spectrophotometry, FT-IR, particle size analysis, and AFM. In the UV–visible spectrophotometry analysis, *Pantoea stewartii* (437 and 351 nm) and *Priestia aryabhattai* (490 nm and 664 nm) showed smooth and narrow absorption peaks. This is the primary confirmation of the presence of silver nanoparticles in the sample. Similarly, Kalaimurugan et al. [40] reported using a UV–visible spectrophotometer to confirm the presence of *P. fluorescens* (YPS3)-synthesized Ag NPs, which exhibit a maximum absorption at 420 nm. According to Soni and Prakash [43], *L. monocytogenes*- (J0161), *B. subtilius*- (ATCC 11774), and *S. anulatus*-synthesized (MTCC 2528) silver nanoparticles showed absorption maxima at wavelengths of 350–750 nm. Encarnacion et al. [44] reported that *E. coli*-synthesized Ag NPs show a narrow UV-vis spectrum absorption peak at 420 nm.

The FT-IR results of *Pantoea stewartii*- and *Priestia aryabhattai*-synthesized silver nanoparticles showed various functional groups from 518.82 cm^−1^ to 3733.93 cm^−1^ and from 517.85 cm^−1^ to 3747.43 cm^−1,^ respectively. The FT-IR results of culture pellets of *Pantoea stewartii* and *Priestia aryabhattai* showed different functional groups from 431.06 cm^−1^ to 3720.43 cm^−1^ and from 651.89 cm^−1^ to 3772.5 cm^−1,^ respectively. Comparing the FT-IR spectra of bacterial pellets and synthesized Ag NPs, groups such as alkenes, alcohols, aromatic amines, nitro compounds, and halo compounds were only present in bacterially synthesized Ag NPs. Kumar et al. [45] used an Alpha interferometer to perform FT-IR analysis to determine the functional groups responsible for capping, reducing, and stabilizing Ag NPs, which revealed peaks from 500 cm^−1^ to 4000 cm^−1^. Lateef et al. [46] characterized the synthesized silver nanoparticles using FT-IR measurements to identify the possible biomolecules responsible for stabilizing the synthesized Ag NPs. Monowar et al. [47] found an absorption peak at 3287.30 cm^−1^ in the produced nanoparticle FT-IR spectra, reflecting protein N−H functional groups and phenol/alcohol OH stretching vibrations. Protein C−O stretching causes a 1643.83 cm^−1^ band. The bands at 1229.20 cm^−1^ and 1072.8 cm^−1^ are C−O stretching vibrations, while the peak at 1389.20 cm^−1^ is the symmetric deformation of the CH_3_ vibration. The analysis of all FT-IR results showed the presence of an amine group, representing a derivative of silver nitrate, and the presence of an alcohol group, which is a waste product formed during the silver reduction process, and we again confirmed the presence of silver nanoparticles in the sample.

In the present study, a particle size analyzer was used to determine the histogram of the DLS of the synthesized Ag NPs. The results show that the particle size of *Pantoea stewartii*-synthesized Ag NPs is 3024 nm. *Priestia aryabhattai*-synthesized silver nanoparticles have a size of 2550 nm, confirming that the isolated microorganisms synthesized them. Likewise, Kalainila et al. [48] reported that a particle size analyzer was used to determine the average particle size, size distribution, and polydispersity index (PDI) of synthesized Ag NPs. The polydispersity index was 0.263, and the average particle diameter was 156 nm. Similarly, According to Bharathi et al. [49], according to the size distribution histogram of DLS, the synthesized Ag NPs had a diameter of 86.84 nm and a polydispersity of 0.231.

AFM was performed for the morphological study of *Pantoea stewartii*- and *Priestia aryabhattai*-synthesized silver nanoparticles. Both bacterially synthesized Ag NPs were circular-shaped and disaggregated with a size distribution of 4 nm (*Pantoea stewartii*) and 3.6 nm (*Priestia aryabhattai*). Shamaila et al. [50] used atomic force microscopy (AFM) to analyze gold samples G1 and G2 for their particle presence, size, and distribution. Histogram analysis showed that 6–34 nm gold NPs (G1) were fabricated. The maximum particle sizes were 15–22 nm. The histogram showed that sample G2 gold NPs were 20–40 nm. Saravanan et al. [51] characterized the bacterial exopolysaccharide synthesis of silver nanoparticles. Nanoparticle measurements were made using an AFM probe analysis. An average of 30 nm was found to be the height of the EPS-Ag NPs, as measured at several sites.

The *Pantoea stewartii*- and *Priestia aryabhattai*-synthesized Ag NPs exhibited potent larvicidal activity against the fourth instars of the important vectors *Ae*. *aegypti*, *An*. *stephensi*, and *Cx*. *quinquefasciatus* exposed to different concentrations (10 μg/mL, 20 μg/mL, 30 μg/mL, 40 μg/mL, and 50 μg/mL) for 24 h. The maximum mortality caused by *Pantoea stewartii*-synthesized Ag NPs was observed for *Cx*. *quinquefasciatus*, with LC_50_ and LC_90_ values of 14.829 and 43.575 µg/mL, followed by *An*. *stephensi* at 20.977 and 62.55 µg/mL and *Ae*. *aegypti* at 30.584 and 106.35 µg/mL, respectively. *Priestia aryabhattai*-synthesized Ag NPs demonstrated potent larvicidal action, with LC_50_ and LC_90_ values of 10.736 and 44.394 µg/mL for *Cx*. *quinquefasciatus*, 14.015 and 66.544 µg/mL for *An*. *stephensi*, and 20.668 and 79.911 µg/mL for *Ae*. *aegypti*, respectively. Significant increases in mortality were observed in a dose-dependent manner as synthesized Ag NPs were added. These results are consistent with silver nanoparticles biosynthesized by *Bacillus thuringiensis*, exhibiting significant toxicity against *Ae. aegypti* at a low lethal dosage of 0.10 ppm [52]. *Cx. Quinquefasciatus* exhibited greater susceptibility to *Pseudomonas aeruginosa* sp.-synthesized Ag NPs and showed 100% mortality after one hour of incubation [53].

After being treated with bacterially synthesized silver nanoparticles, larval body parts were subjected to histopathological analysis, which helps to view the affected body parts. The epithelial cells, food bolus, basement membrane, muscles, and midgut were damaged. The damage to the larval bodies depended on the concentration of nanoparticles. The microscopic and histological examination results show that *Pantoea stewartii*- and *Priestia aryabhattai*-synthesized Ag NPs caused damage to the midgut, muscles, basement membrane, and numerous portions of the feeding bolus of the fourth-instar larvae of *Ae. aegypti*, *An. stephensi*, and *Cx. quinquefasciatus*. This differentiates the injured larvae from the control larvae.

Suganya et al. [54] reported the histopathological analysis of third-instar larvae of *Ae. aegypti* treated with HAuCl_4_. Damaged epithelial cell layers and even damaged epithelial cells were evident in the larvae. Additionally, *Ae. aegypti* treated with Ze-Au NPs exhibited the total disintegration of the abdominal region, particularly the midgut and caeca, resulting in the loss of antenna hairs, lateral hairs, caudal hairs, and upper and lower head hairs. The abdominal region of *Ae. aegypti* treated with Ze-Au NPs also showed a significant decrease. The midgut epithelial cells of treated and control larvae exhibited different histological structures, according to investigations by Fouad et al. [55]. After being exposed to rhizome-fabricated Ag NPs, the midgut cells of *Ae. aegypti* larvae in their fourth-instar stage showed varying degrees of apical swelling into the gut lumen, decreased intercellular contacts, and nuclei and brush border degeneration. Kalimuthu et al. [56] also reported the histopathological analysis of Ag-NP-treated mosquito larvae. The most visible effect on the mosquito larval body damage was the larval midgut, and the epithelial cells, midgut lumen, and head and tail parts were also majorly affected.

In the present work, the harmful effects of different concentrations of *Pantoea stewartii*- and *Priestia aryabhattai*-synthesized Ag NPs on brine shrimp *Artemia nauplii* growth, survival, and mortality under intermittent flow-through conditions were evaluated to determine non-target effects. *Artemia nauplii* were treated with various concentrations of Ag NPs, namely, 10 µg/mL, 20 µg/mL, 40 µg/mL, 60 µg/mL, and 90 µg/mL, for 48 h. The findings demonstrated that the animals in the control group exhibited no signs of aggregation. No mortality was observed in the control or in groups treated with *Pantoea stewartii*-mediated Ag NPs at lower concentrations (10 and 20 µg/mL). The LC_50_, LC_90_, and χ2 values were 63.44 (38.484–104.579), 157.095 (95.297–258.968), and 0.619 µg/mL. No mortality was observed in the control or in groups treated with *Priestia aryabhattai*-mediated Ag NPs at lower concentrations (10 and 20 µg/mL), but 67% mortality was observed at higher concentrations (90 µg/mL). The LC_50_ and LC_90_ values were 74.595 (42.455–131.065) and 206.893 (117.752–363.517). *Pantoea stewartii*- and *Priestia aryabhattai*-mediated Ag NPs were less toxic (less than 50%) to the non-target species *A*. *nauplii* than to mosquitoes. Similarly, Arulvasu et al. [57] investigated the detrimental impact of different concentrations of silver nanoparticles on the growth of brine shrimp *Artemia nauplii*. About 10 nauplii were treated with the appropriate concentrations of Ag NPs, i.e., 2 nM, 4 nM, 6 nM, 8 nM, 10 nM, and 12 nM. The mortality was 16% at a concentration of 2 nM, increasing to around 33%, 36.6%, and 43% at 4 nM, 6 nM, and 8 nM. The percentage of dead *Artemia nauplii* increased by around 53.3% and 66.6% as the concentration increased to 10 nM and 12 nM, respectively. The LC_50_ value was found at a concentration of about 10 nM. Suganya et al. [54] investigated the histopathological effects of the Zika virus vector *Ae. aegypti*, which included the entire disintegration of the abdominal area, particularly in the midgut and caeca, with the loss of lateral and caudal hairs, and stereomicroscopic observations revealed losses of head hairs (upper and lower), antenna, and caudal hairs.

The antimicrobial activity was evaluated using *Pantoea stewartii*- and *Priestia aryabhattai*-synthesized Ag NPs against human pathogenic bacteria and fungi. The maximum inhibitory zones for synthesized Ag NPs against MTCC-1537 *Streptomyces varsoviensis* (1.2 cm), MTCC-160 *Penicillium chrysogenum* (1.2 cm), MTCC-1688 *Pseudomonas aeruginosa* (0.9 cm), MTCC-3160 *Staphylococcus aureus* (1.3 cm), MTCC-733 *Salmonella enterica serovar typhi* (1.0 cm), MTCC-1973 *Aspergillus flavus* (1.2 cm), MTCC-451 *Vibrio parahaemolyticus* (1.0 cm), and MTCC-43 *Escherichia coli* (1.5 cm) were found at a concentration of 80 µL of *Pantoea stewartii*-synthesized Ag NPs. *Priestia aryabhattai*-synthesized Ag NPs exhibited the maximum zones of inhibition against MTCC-1537 *Streptomyces varsoviensis* (1.3 cm), MTCC-160 *Penicillium chrysogenum* (1.0 cm), MTCC-655 *Streptococcus pneumonia* (1.2 cm), and MTCC-3160 *Staphylococcus aureus* (0.3 cm) at a concentration of 80 µL. Similarly, Dar et al. [58] reported the antimicrobial activity of silver nanoparticles synthesized by *Cryphonectria* sp. against three human pathogenic bacteria, *S*. *aureus* (ATCC-25923), *S*. *typhi* (ATCC-51812), and *E*. *coli* (ATCC-39403), and they observed that the Ag NPs showed remarkable antimicrobial activity against *Staphylococcus aureus* (7 mm), *Salmonella typhi* (6 mm), *Escherichia coli* (8 mm), and *Candida albicans* (6 mm). The results of a zone-of-inhibition assay showed that the synthesized Ag NPs were highly effective antimicrobials against a group of common pathogens.

Concentration-dependent toxicity testing was used to determine the embryonic toxicity (10, 20, 30, and 50 µg/mL) of *Pantoea stewartii*- and *Priestia aryabhattai*-synthesized Ag NPs in zebrafish embryos. The highest concentration of both bacterial species’ synthesized Ag NPs showed a slight effect on developing embryos (50 µg/mL) in the presence of the chorion. No incidence of mortality was observed at lower concentrations. At 50 µg/mL, Ag NPs were found to cause non-malformed yolk sac edema in approximately 70% of embryos. *Pantoea stewartii*- and *Priestia aryabhattai*-mediated Ag NPs showed lower toxicity in the non-target zebrafish embryos than in mosquitoes. Similarly, Asharani et al. [59] reported on silver-nanoparticle-treated zebrafish embryos using 50 µg/mL. They observed that the embryo’s chorion revealed the presence of brownish flakes. They reported that 60–90% of the surviving embryos exhibited body abnormalities at a dose of 50 µg/mL. The acute effects of Ag NPs on oocytes were described by Lee et al. [60], and exposure at the larval stage (96–100 hpf) was used to assess embryonic toxicity. In 1 well of a 24-well plate, ten 96-hour-old (hpf) embryos were placed. As a result, oocyte densities fell precipitously in the 1 mg/L and 3 mg/L groups by 25% (*p* = 0.02) and 55% (*p* = 0.001), respectively. There was no increase in the rate of deformed ionocytes in the control group, but there was a significant rise in the 1 and 3 mg/L groups (from 0% to 26% and 54%, respectively; *p* = 0.001). They observed substantial malformation in the embryo. An estimated 70% of embryos exposed to Ag NPs at 50 µg/mL exhibited normal development and no signs of deformity in the yolk sac. Based on the results, bacterially synthesized silver nanoparticles do not affect the non-targeted animals.

## 4. Materials and Methods

### 4.1. Collection of Soil Sample

The soil sample was collected from the Western Ghats—Shenbagathoppu (Latitude: 9:32:42.47; Longitude—77:33:27.59; Altitude—98.263). Using a sterile scoop, soil samples were taken from a depth of 10 cm, transported to the lab in sterile polyethylene bags, and then kept at 20 °C in an icebox for further examination.

### 4.2. Mosquito Larvae

The ICMR Center for Research in Medical Entomology (VCRC) provided the *Aedes aegypti* egg raft and *Culex quinquefasciatus* and *Anopheles stephensi* larvae, which were transported to the lab safely without causing any disturbance. These larvae were kept in deionized-water-filled enamel trays for 24 h before the experiment’s start, during which they were fed a 3:1:1 mixture of brewer’s yeast, dog biscuits, and sugar.

### 4.3. Bacterial Isolation and Characterization

The soil sample was serially diluted and then plated on nutrient agar. Purified bacteria were isolated by repeated quadrant streaking on a nutrient agar medium. The bacterial isolates were examined for their morphological characterization, including colony size, elevation, shape, surface, color or pigmentation, motility, etc. Further, they were characterized by specific microscopic and biochemical tests described by Cappuccino and Sherman [61]. The pure bacterial colonies were obtained, and the isolates were denoted as H2 and H3.

### 4.4. Bacterial Characterization Using Molecular Techniques

Microbes were identified, categorized, and quantified by sequencing their 16S rRNA genes. Using a QIAamp DNA Mini Kit, bacterial DNA was extracted, and then PCR was used to amplify 16S rRNA using 17F and 1492R primers. The PCR product was purified with a Bionteq Gel Elution Kit.

### 4.5. Intracellular Synthesis of Silver Nanoparticles

Separate H2 and H3 isolates were inoculated into a nutrient broth, and the mixture was shaken for 48 h. The bacterial growth medium was centrifuged at 4000 rpm for 20 min after incubation, obtaining a pellet and a supernatant. Then, the pellet was resuspended in distilled water, and 1 mM silver nitrate was added [62]. It was then incubated overnight at 60 °C and pH 10 [11]. After incubation, dark brown color formation was observed (i.e., indicating the synthesis of nanoparticles).

### 4.6. Washing and Purification of Silver Nanoparticles

This step was performed following the procedure described by Rai and Duran [63]. The bacterial cell suspension holding the nanoparticles was centrifuged at 8000 rpm for 10 min at room temperature. The particles were centrifuged for 10 min after being resuspended in NaCl solution. The procedure described above was carried out three times to be absolutely confident that nothing undesirable was left behind. A mortar was used to crush the pellets, and then egg-white lysozyme was added to the mixture to break up the cells. The pellets were centrifuged and resuspended in a complete salt solution (pH 7.5) containing NaCl (17.5 g/L), KCl (0.74 g/L), MgSO_4_.7H_2_O (12.3 g/L), and Tris HCl (0.15 g/L). The remaining cells were incubated in a complete salt solution containing 20 mg of egg-white lysozyme at 22 °C for 18 h. The lysed mixture was removed from the nanoparticles by washing them with a complete salt solution and distilled water and centrifuging the particles at 4000 rpm for 20 min. The nanoparticles were resuspended in distilled water and placed in a Petri dish to dry in an oven preheated to 80 °C. The dried particles were collected for further analysis.

### 4.7. Characterization of Silver Nanoparticles

#### 4.7.1. UV–Visible Spectroscopy Analysis

The reaction mixture was subjected to observation at different wavelengths to confirm the silver nanoparticles’ formation. Absorption maxima were scanned with a UV-vis spectrophotometer between 325 and 825 nm on aqueous component samples to confirm the presence of the bacterially synthesized nanoparticles (Shimadzu UV 1700).

#### 4.7.2. FT-IR Spectroscopy

A Fourier transform infrared spectrophotometer was used to characterize the biosynthesized nanoparticles. The Shimadzu 8400S FT-IR spectrometer was used to compare the IR spectra of the bacterial product to a standard, with the peak value between 400^−1^ and 500 cm^−1^ providing insight into the functional groups of molecules [64].

#### 4.7.3. Atomic Force Microscopy

The biosynthesized Ag NPs were characterized by atomic force microscopy (AFM). It was used to characterize silver nanoparticles’ 3D profiles, dispersity, and mean particulate sizes [65].

### 4.8. Larvicidal Bioassay

The biologically synthesized Ag NPs were tested for their ability to kill mosquito larvae using a method described by WHO [7]. Ten mosquito larvae in the fourth-instar stage were placed in disposable cups with 200 mL of distilled water and a 250 mL capacity. Three replicates with 30 larvae were carried out for each concentration. Larval mortality was recorded 24 h after exposure, during which the larvae were not fed. Parallel to each replication, control tests were also carried out. The appropriate container was filled with various concentrations of silver nanoparticles (10, 20, 30, 40, and 50 μg/mL), and the larval behavior was monitored after 24 h. Mosquito larval behavior changed after exposure to the biosynthesized nanoparticles, showing slow swimming, standing in a single place, changes in larval color, damaged body parts, and mortality. Statistics were compiled from the fatalities that occurred.

### 4.9. Histopathological and Stereomicroscopic Analysis

Histological analysis of silver-nanoparticle-exposed mosquito larvae was performed. A 10% buffered formaldehyde solution was used to store the afflicted larvae for 24 h before they were dehydrated using an ethanol series and finally cleaned using xylene solutions. At the embedding stage, melted paraffin was used to embed them inside the block permanently. Hematoxylin and eosin were used to stain the paraffin blocks, and sections were cut from them at a thickness of 5 m using a rotary microtome. The effects of the extract’s toxicity on larvae were observed at a magnification of 40× under a bright-field light microscope. Under a stereomicroscope, the extract-induced accumulation of tissue and its modifications were observed [66].

### 4.10. Antimicrobial Activity of Bacterially Synthesized Silver NPs

The agar well diffusion method was used to determine the antimicrobial activity of *Bacillu saryabhattai* (H3)- and *Pantoea stewartii* (H2)-synthesized Ag NPs. Seven bacterial strains and two fungal strains were selected for antimicrobial activity studies. *Streptomyces varsoviensis* MTCC-1537, *Streptococcus pneumoniae* MTCC-655, *Penicillium chrysogenum* MTCC-160, *Staphylococcus aureus* MTCC-3160, *Pseudomonas aeruginosa* MTCC-1688, *Salmonella enterica serovar typhi* MTCC-733, *Aspergillus flavus* MTCC-1973, *Vibrio parahaemolyticus* MTCC-451, and *Escherichia coli* MTCC-43 were used as the test organisms. All of these isolates were obtained from Microbial Type Culture Collection, Pune, India. LB agar was used for all subsequent subculturing of microorganisms (Himedia, Mumbai, India). The microorganisms were then cultivated in a rotating shaker incubator at 37 °C in a 100 mL Erlenmeyer flask with a nutrient broth medium (Himedia, Mumbai, India). After centrifuging the culture, collecting the pellet, and washing it twice with water and once with Phosphate-Buffered Saline (PBS), the broth was diluted to the desired concentration (106 CFU/mL). To determine the antimicrobial activities of the green synthesized NPs, different concentrations (3, 5, and 8 µgmL^−1^) and an 8 µgmL^−1^ standard of chloramphenicol were loaded into the wells. The plate was placed in the refrigerator for around two hours for sample diffusion. The antibacterial activity was evaluated and compared to the standard after all plates were incubated at 37 °C overnight. The inhibitory zones were measured [67].

### 4.11. Toxicity Assay on Artemia salina

#### 4.11.1. Cultivation of *Artemia salina*

The cysts were decapsulated for 15 min with sodium hypochlorite before the assay. *Artemia nauplii (A. nauplii)* cysts were hatched in salt water (30% m/v) by dissolving the necessary amount of salt in deionized water and agitating the mixture for 36 h while maintaining adequate oxygen levels. A 30 μm Millipore filter was used to purify it. Encysted *A. nauplii* were rehydrated in distilled water at 4 °C for 12 h, after which the floating cysts were removed from the conical flask and cleaned. After being cleaned in cold deionized water, the cysts were collected using a Buchner funnel as they sank. In a conical flask at 30 ± 3 °C (A1500 lux daylight consistently supplied by a fluorescent lamp), 2 g of the pre-cleaned cysts was added to 1.0 L of saltwater. At the same time, aeration was maintained at a constant rate (a small line extending to the bottom of the hatching device from an aquarium air pump). *A. nauplii* started to hatch after 36 h [66].

#### 4.11.2. Non-Target Bioassay on *Artemia nauplii*

Under intermittent flow-through conditions, the growth, survival, and death of the brine shrimp Artemia nauplii were measured to determine the acute toxicity of silver nanoparticles. The experiment started with nauplii that were 24 h old and continued for another 24 and 48 h with the same exposure conditions. A 12-well plate was used for the experiment. About ten nauplii were placed into each well containing 2 mL of 33 ppt saline water with the addition of the desired concentrations of Ag NPs (10, 20, 40, 60, and 90 µg/mL). In a 12-well plate, three replicates of each concentration were tested. The experimental setup was left in the dark for 24 h, and the number of *A. nauplii* was counted afterward. After 24 h, the LC_50_ value and percentage of mortality were calculated for each test concentration of silver nanoparticles and compared to the control.

#### 4.11.3. Embryonic Zebrafish Non-Target Assay

Collected embryos were staged and dechlorinated with machine assistance at four hours post-fertilization (hpf). Ag NPs with a concentration of 1% were made by first diluting particles in a series of dilutions. A Pasteur pipette was used to transfer six dechlorinated, high-passage-focal-length embryos into each well of a 96-well plate containing 90 µL of the test medium. Once embryos were placed in the plate, 10 L of the 1% nanoparticle solution was diluted to final concentrations of 10, 20, 30, 40, and 50 ppm and added to each well. At 24 and 120 h post-fertilization (hpf), exposed embryos were examined for 22 different outcomes. Mortality rates during embryonic development were measured at 24 and 120 h post-fertilization. Each treatment’s proportion of deformity was determined by the ratio of afflicted to viable embryos. Each component of EM was tested to determine the conditions that maintained zebrafish embryo health and Ag NP stability. Zebrafish embryos were exposed to silver nanoparticles as a positive control for exposure to silver ions. The activity and growth level of the embryo were observed under the microscope [68]. In the individual toxicity tests of zebrafish, LC_50_ and LC_90_ values, 95% confidence intervals (CIs), and chi-square were calculated.

### 4.12. Statistical Analysis

The LC_50_ and LC_90_ values were calculated using probit analysis. Using SPSS version 16.00, the fiducial limit (FL) was determined with a 95% confidence limit (CL) and the chi-square (χ^2^) value, where a p-value less than 0.05 was considered statistically significant.

## 5. Conclusions

The present study suggests that the green synthesized Ag NPs from *Pantoea stewartii* (H2) and *Priestia aryabhattai* (H3) showed good larvicidal activity against mosquito larvae and lower toxicity against non-target organisms. Hence, the synthesized Ag NPs will serve as a perfect substitution for chemical larvicides and be a low-cost and eco-friendly approach. Further studies assessing the stabilization of silver nanoparticles in water and adverse effects on other organisms in water bodies would pave the way to the construction of a nan device to manage the mosquito population and prevent mosquito-borne diseases.

## Figures and Tables

**Table 1:** Larvicidal activity of *Pantoea stewartii*-mediated Ag NPs against 4th-instar larvae of major mosquito species.

Mosquito Larval Species n^a^ (150)	Concentration of Ag NPs (µg/mL)	% Mortality	LC_50_ LUL-UCL (µg/mL)	LC_90_ LUL-UCL (µg/mL)	Intercept	χ^2^	Regression Equation
*Ae.* *aegypti*	10	17	30.584 17.07–54.78	106.35 59.37–190.53	−3.517	0.762	Y = −0.09 X = 28.01
	20	30					
	30	40					
	40	57					
	50	80					
	Control	0					
*An.* *stephensi*	10	30	20.977 12.39–35.48	62.55 36.97–105.82	−3.57	0.34	Y = 0.236 X = 25.64
	20	30					
	30	63					
	40	73					
	50	97					
	Control	0					
*Cx.* *quinquefasciatus*	10	37	14.829 8.54–25.75	43.575 25.09–75.68	−3.206	0.864	Y = 0.516 X = 22.88
	20	57					
	30	77					
	40	87					
	50	97					
	Control	0					

(n^a^—Total number of mosquitoes used per species: 10 per replicate, with three replicates carried out and five concentrations tested; LC_50_—lethal concentration killing 50% of exposed organisms; LC_90_—lethal concentration killing 90% of exposed organisms; LCL—95% lower confidence limits; UCL—95% upper confidence limits; χ^2^—chi-square).

**Table 2:** Larvicidal activity of *Priestia aryabhattai*-mediated Ag NPs against 4th-instar larvae of major mosquito species.

Mosquito Larval Species n^a^ (150)	Concentration of Ag NPs (µg/mL)	% Mortality	LC_50_ LUL-UCL (µg/mL)	LC_90_ LUL-UCL (µg/mL)	Intercept	χ^2^	Regression Equation
*Ae.* *aegypti*	10	33	20.668 10.98–38.88	79.911 42.47–150.34	−2.87	0.507	Y = 0.207 X = 25.705
	20	40					
	30	53					
	40	70					
	50	93					
	Control	0					
*An.* *stephensi*	10	47	14.015 6.65–29.52	66.544 31.58–140.20	−2.172	0.649	Y = 0.45 X = 24.232
	20	53					
	30	63					
	40	83					
	50	93					
	Control	0					
*Cx.* *quinquifasciatus*	10	53	10.736 5.18–22.22	44.394 21.44–91.91	−2.143	0.735	Y = 0.672 X = 22.6
	20	67					
	30	73					
	40	90					
	50	97					
	Control	0					

(n^a^—Total number of mosquitoes used per species: 10 per replicate, with three replicates carried out and five concentrations tested; LC_50_—lethal concentration killing 50% of exposed organisms; LC_90_—lethal concentration killing 90% of exposed organisms; LCL—95% lower confidence limits; UCL—95% upper confidence limits; χ^2^—chi-square).

**Table 3:** Antimicrobial activity of *Priestia aryabhattai* (H3)-synthesized Ag NPs against selected human pathogens.

S. No.	Microbial Isolates	Control	Zone of Inhibition (cm)		
			30 μL	50 μL	80 μL
1.	*Streptomyces varsoviensis* (MTCC-1537)	1.8	1.0	1.2	1.3
2.	*Penicillium chrysogenum* (MTCC-160)	1.4	0.5	0.7	1.0
3.	*Streptococcus pneumoniae* (MTCC-655)	1.8	0.7	1.0	1.2
4.	*Staphylococcus aureus* (MTCC-3160)	0.8	-	0.1	0.3

**Table 4:** Antimicrobial activity of *Pantoea stewartii* (H2)-synthesized Ag NPs.

S. No.	Microbial Isolates	Zone of Inhibition (cm)			
		Control	30 μL	50 μL	80 μL
1.	MTCC-1537 *Streptomyces varsoviensis*	1.7	1.0	1.0	1.2
2.	MTCC-160 *Penicillium chrysogenum*	1.6	1.1	1.1	1.2
3.	MTCC-1688 *Pseudomonas aeruginosa*	1.4	0.4	0.8	0.9
4.	MTCC-3160 *Staphylococcus aureus*	1.6	1.0	1.0	1.3
5.	MTCC-733 *Salmonella enterica serovar typhi*	1.7	0.7	0.8	1.0
6.	MTCC-1973 *Aspergillus flavus*	2.0	0.9	1.0	1.2
7.	MTCC-451 *Vibrio parahaemolyticus*	0.8	0	0.1	0.2
8.	MTCC-43 *Escherichia coli*	1.5	1.0	1.2	1.5

**Table 5:** Toxicity assay of *Pantoea stewartii*-mediated Ag NPs on *Artemia nauplii*.

Concentration of Ag NPs (µg/mL)	Total No. of *Artemia* Exposed	% Mortality	LC_50_ (LCL-UCL) µg/mL	LC_90_ (LCL-UCL) µg/mL	χ^2^	Regression Equation
10	10	0	63.44 38.48–104.58	157.095 95.30–258.97	0.619	Y = −0.291 X = 51.634
20	10	0				
40	10	27				
60	10	37				
90	10	77				
Control	10	0				

LC_50_—lethal concentration killing 50% of exposed organisms; LC_90_—lethal concentration killing 90% of exposed organisms; LCL—95% lower confidence limits; UCL—95% upper confidence limits; χ^2^—chi-square.

**Table 6:** Toxicity assay of *Priestia aryabhattai*-mediated Ag NPs on *Artemia nauplii*.

Concentration of Ag NPs (µg/mL)	Total No. of *Artemia* Exposed	% Mortality	LC_50_ (LCL-UCL) µg/mL	LC_90_ (LCL-UCL) µg/mL	χ^2^	Regression Equation
10	10	0	74.595 42.45–131.06	206.893 117.75–363.51	0.711	Y = −0.448 X = 52.218
20	10	0				
40	10	23				
60	10	30				
90	10	67				
Control	10	0				

LC_50_—lethal concentration killing 50% of exposed organisms; LC_90_—lethal concentration killing 90% of exposed organisms; LCL—95% lower confidence limits; UCL—95% upper confidence limits; χ^2^—chi-square.

**Table 7:** Toxicity assay of *Pantoea stewartii*-mediated Ag NPs on embryos.

Concentration of Ag NPs (µg/mL)	Total No. of Embryos Exposed	% Mortality	LC_50_ (LCL-UCL) µg/mL	LC_90_ (LCL-UCL) µg/mL	χ^2^	Regression Equation
10	10	0	299.187 76.00–1177.70	2038.019 517.748–8022.291	0.92	Y = −1.469 X = 33.186
20	10	0				
30	10	10				
50	10	14				
Control	10	0				

LC_50_—lethal concentration killing 50% of exposed organisms; LC_90_—lethal concentration killing 90% of exposed organisms; LCL—95% lower confidence limits; UCL—95% upper confidence limits; χ^2^—chi-square.

**Table 8:** Toxicity assay of *Priestia aryabhattai*-mediated Ag NPs on embryos.

Concentration of Ag NPs (µg/mL)	Total No. of Embryos Exposed	% Mortality	LC_50_ (LCL-UCL) µg/mL	LC_90_ (LCL-UCL) µg/mL	χ^2^	Regression Equation
10	10	0	101.032 47.53–214.74	331.537 155.98–704.66	0.716	Y = −1.13 X = 35.437
20	10	0				
30	10	20				
50	10	25				
Control	10	0				

LC_50_—lethal concentration killing 50% of exposed organisms; LC_90_—lethal concentration killing 90% of exposed organisms; LCL—95% lower confidence limits; UCL—95% upper confidence limits; χ^2^—chi-square.

## Data Availability

Not applicable.
